# The Role of Mesostriatal Dopamine System and Corticostriatal Glutamatergic Transmission in Chronic Pain

**DOI:** 10.3390/brainsci11101311

**Published:** 2021-10-02

**Authors:** Barbara Ziółkowska

**Affiliations:** Department of Molecular Neuropharmacology, Maj Institute of Pharmacology, Polish Academy of Sciences, 12 Smętna Str., 31-343 Kraków, Poland; nfziolko@cyf-kr.edu.pl; Tel.: +48-12-66-23-238

**Keywords:** pain, dopamine, striatum, nucleus accumbens, corticostriatal, medial prefrontal cortex

## Abstract

There is increasing recognition of the involvement of the nigrostriatal and mesolimbic dopamine systems in the modulation of chronic pain. The first part of the present article reviews the evidence indicating that dopamine exerts analgesic effects during persistent pain by stimulating the D2 receptors in the dorsal striatum and nucleus accumbens (NAc). Thereby, dopamine inhibits striatal output via the D2 receptor-expressing medium spiny neurons (D2-MSN). Dopaminergic neurotransmission in the mesostriatal pathways is hampered in chronic pain states and this alteration maintains and exacerbates pain. The second part of this article focuses on the glutamatergic inputs from the medial prefrontal cortex to the NAc, their activity changes in chronic pain, and their role in pain modulation. Finally, interactions between dopaminergic and glutamatergic inputs to the D2-MSN are considered in the context of persistent pain. Studies using novel techniques indicate that pain is regulated oppositely by two independent dopaminergic circuits linking separate parts of the ventral tegmental area and of the NAc, which also interact with distinct regions of the medial prefrontal cortex.

## 1. Introduction

Chronic pain is a common condition, which affects nearly 20% of people in Western societies. Thirty to fifty percent of them experience severe and/or persistent pain, which seriously affects their everyday functioning [1,2,3]. The increasingly widespread use of prescription opioids, the most potent analgesic drugs, has led to an epidemic of synthetic opioid abuse [4] while leaving the needs of many chronic pain sufferers unmet [5]. The frequently observed resistance of chronic pain to conventional analgesic treatment [6,7] is most likely due to maladaptive plastic changes in the central nervous system (CNS), which develop in response to prolonged nociceptive stimulation resulting from an initial injury, inflammation, or other diseases that damage peripheral tissues and/or parts of the somatosensory nervous system. Such morbid neural plasticity is probably responsible for the continuation of painful sensations even after the initial lesion has healed, underlying the occurrence and persistence of such symptoms as spontaneous pain, hyperalgesia (increased sensitivity to nociceptive stimuli), and allodynia (pain in response to stimuli which are normally innocuous, e.g., touch) [8]. Even though there has been major progress in elucidating the mechanisms of nociception, and a large number of molecular alterations accompanying chronic pain have been found in the spinal cord [9,10,11], the existing methods of chronic pain pharmacotherapy are still inadequate. Therefore, there is an urgent need for a better understanding of the CNS mechanisms underlying chronic pain, both at the system level (i.e., identifying functionally crucial CNS regions) and the molecular level (identifying potential drug targets).

## 2. Models and Behavioral Testing of Pain in Animals

Recent studies in humans, using brain imaging techniques, have brought important findings regarding the brain regions involved in and factors predisposing to chronic pain development [12]. However, insights into the cellular and molecular nature of changes responsible for the persistence of pain rely largely on animal experiments. An extensive overview of models and behavioral tests used in studies of chronic pain in rodents can be found in the articles by Burma et al. [13], and Coderre and Laferrière [14]. In brief, the most commonly used rodent models of inflammatory pain involve injections of inflammation-producing substances such as the complete Freund’s adjuvant or carrageenan into the plantar region or a joint of the hind paw. Neuropathic pain, i.e., pain elicited by a lesion or disease of the somatosensory nervous system [8], is usually modeled by chronic constriction or partial transection of the sciatic nerve. These lesions produce long-lasting hypersensitivity of the affected paw to noxious and innocuous stimuli, reflected by a decrease of nociceptive thresholds. In most studies, the lesions are performed unilaterally, so that the unaffected contralateral limb can serve as a control in sensory sensitivity experiments. Apart from the variations of those two types of models, there also exist rodent paradigms that model specific pain disorders, e.g., migraine and cancer pain (cf [13,14]).

Not all aspects of pain are easy to assess in animals. Chronic pain has a somatosensory component, which includes spontaneous pain and hypersensitivity to mechanical, thermal, and chemical stimuli (hyperalgesia and allodynia), as well as an emotional component involving aversion elicited by the sensory perception of pain, which is often accompanied by depression-like states (decreased motivation, anhedonia) and anxiety. On the somatosensory spectrum, only the sensory hypersensitivity is straightforward to measure by assessing reflexive responses to acute somatosensory stimulation. In contrast, there is no objective measure of ongoing pain. Its presence can be inferred from place conditioning experiments aimed at detecting aversion associated with pain and conditioned place preference (CPP) of environments associated with pain relief (e.g., places where analgesic drugs were administered) [15].

Behavioral tests most commonly used to gauge affective changes associated with pain include those reflecting depression-like states (forced swim test, tail suspension test), anhedonia (consumption of sucrose solution), anxiety (e.g., open field test or elevated plus maze), social interactions, and motivation (e.g., instrumental self-administration of palatable food on a progressive ratio schedule) (cf [16]).

## 3. The Mesostriatal Dopamine System. Brain Imaging and Inactivation Experiments Implying Its Involvement in Pain Processing 

Accumulating evidence indicates that pain processing strongly involves forebrain regions that do not belong to the somatosensory system, particularly the prefrontal cortex, amygdala, and striatum [12,17]. The activity of neural circuits which include the above-mentioned interconnected parts of the brain is potently modulated by dopaminergic projections from the midbrain to the striatum. This mesostriatal dopamine system consists of (1) projections from the substantia nigra pars compacta (SNc) to the dorsal striatum (dStr) (also referred to as the nigrostriatal pathway), which are involved in the regulation of motor functions by the extrapyramidal system and (2) projections from the ventral tegmental area (VTA) to the ventral striatum, i.e., mainly the nucleus accumbens (NAc) (also referred to as the mesolimbic pathway), which were classically considered as the core of the brain reward system, responsible for signaling reward and reward learning, but which in fact code the salience and valence not only of rewarding but also aversive stimuli. The latter connections are segregated in such a manner that the medial part of the VTA innervates the medial NAc shell (mNAc shell), whereas the lateral VTA innervates the lateral NAc shell and the NAc core [18,19,20].

Changes in perception of painful stimuli were demonstrated in humans having lesions within the dStr [21] and in animals following inactivation of the NAc or the SNc with local anesthetics [22,23]. In patients suffering from chronic pain conditions, morphological abnormalities were detected in the striatum, such as a decreased grey matter density in the dStr and NAc as well as reduced NAc volume [24,25,26]. In addition, functional brain imaging in chronic pain patients showed alterations in functional connectivity between the NAc and the medial prefrontal cortex (mPFC) [12,25,26,27]. Longitudinal studies in patients transitioning from subacute to persistent back pain demonstrated that some of these structural and functional changes develop only when the pain becomes chronic (decreases in grey matter density, alterations of the NAc activity), whereas others pre-exist and apparently predispose some individuals to chronification of pain states (small volume of the NAc, strong functional connection of the NAc to mPFC) [24,26]. Moreover, the strength of the NAc to mPFC functional connectivity correlates with the reported intensity of chronic pain in humans [27]. The above observations implicate the components of the mesostriatal pathways, as well as the related parts of the frontal cortex, in pain processing and its transition to a persistent state. Neurotransmission mechanisms underlying these processes involve actions of the mesostriatal dopamine in individual striatal subregions and their interplay with the excitatory corticostriatal input. 

## 4. The Effect of Nociceptive Stimuli on Dopaminergic Neurons

Even though initial studies demonstrated that some mesencephalic dopaminergic neurons were inhibited by painful stimuli [28], further research indicated that acute nociceptive stimulation activates a portion of these cells [29]. Consequently, painful stimulation increases dopamine release in the dStr and NAc core, as suggested by the evidence from both rodent and human studies, whereas it may suppress dopamine neurotransmission in the NAc shell [30,31,32,33]. In people with no chronic pain conditions, the amount of dopamine released in response to a nociceptive stimulus correlates with the intensity of perceived pain [32,33]. The dopamine response in the NAc in humans is associated only with the emotional perception of pain; in contrast, the response in the dStr is correlated with both sensory and affective pain ratings [32].

## 5. The Effect of Dopamine Deficiency on Nociception and Pain

Observations in humans and animals suggest that dopamine in the striatum exerts antinociceptive effects, whereas its deficiency promotes pain. Thus, chronic pain is a frequent symptom in Parkinson’s disease (PD), the epitome of mesostriatal dopamine loss in man. Present in up to 85% of patients, the pain symptoms of PD are of varied origin, some of them being considered as central (primary) pain, not related to any physical disturbances. This PD-related pain is alleviated by the dopamine precursor L-DOPA or dopamine receptor agonists [34,35]. Moreover, PD patients show hyperalgesia, i.e., increased sensitivity to acute nociceptive stimuli, and decreased emotional tolerability of painful stimulation [36,37]. Meta-analyses by Sung et al. [38] demonstrated that the PD-associated hyperalgesia is relieved shortly after the administration of dopaminergic drugs (as compared to the medication-off state after an overnight withdrawal). They also showed that the sensitivity to evoked pain is greater in the PD patients who experience chronic pain than in those who do not [38].

Similarly, 6-hydroxydopamine (6-OHDA) lesions of the mesostriatal dopamine system in rodents, which serve as a model of PD, lead to a decrease in nociceptive thresholds in response to mechanical, thermal, and chemical stimuli [39,40,41,42,43], aggravate chronic neuropathic pain [42,44] and produce allodynia on the snout lasting for many weeks [44,45]. Decreased nociceptive thresholds have also been observed in another commonly used PD model, i.e., in mice treated with the neurotoxin 1-methyl-4-phenyl-1,2,3,6-tetrahydropyridine (MPTP), where some of the changes in nociceptive sensitivity were reversed by L-DOPA [46,47]. Moreover, destroying the ventral mesostriatal DA system with 6-OHDA led to attenuation of morphine analgesia in the formalin test and, to a lesser extent, in the tail-flick test [48].

Altogether, these observations indicate that hypodopaminergic states of PD and those after experimental lesions of the mesostriatal dopamine pathway promote chronic pain as well as acute hyperalgesia. Those symptoms are reversed by dopaminomimetic drugs, and there is an association of dopamine loss-related hyperalgesia with the propensity to develop chronic pain in humans.

## 6. The Role of Striatal Dopamine Receptors in Pain Regulation

Evidence accumulated since the 1970s has demonstrated that electrical stimulation of the VTA or the substantia nigra increases nociceptive thresholds and exerts analgesic effects in inflammatory pain [41,49,50,51]. These observations were extended by the recent studies using opto- and chemogenetic approaches which showed that stimulation of identified VTA dopaminergic neurons or their terminals in the NAc exerted an acute antinociceptive effect as well as reduced thermal hyperalgesia in chronic inflammatory, neuropathic, and cancer pain models [52,53]. Physiologically, the critical role in controlling dopaminergic cells activity during pain has been ascribed to the rostromedial tegmental nucleus (RMTg). RMTg, a GABAergic structure adjacent to the VTA, has an inhibitory influence on dopaminergic neurons [52,54,55,56]. As recently demonstrated by Taylor et al. [52], relieving that inhibition elicited dopamine release in the NAc, which resulted in strong analgesia both in acute and chronic pain paradigms. RMTg, rich in the mu-opioid receptors, is also the key mediator of analgesic actions of opioids, which involve the same dopaminergic mechanism [52,55,57].

The dopamine influence on pain was studied further by assessing the effects of dopamine receptor ligands injected locally into the striatum. Administration of nonselective dopaminomimetic drugs (e.g., amphetamine, apomorphine) or selective dopamine D2 receptor (D2R) agonists (quinpirole) into the dStr or NAc raised thermal nociceptive thresholds and conferred analgesia in neuropathic pain, as well as in tonic pain elicited by subcutaneous injection of formalin or prostaglandin E2 [41,58,59,60,61]. On the other hand, D2R antagonists (eticlopride, raclopride, haloperidol) administered into the dStr, NAc or intracerebroventricularly, reversed antinociceptive effects of dopamine agonists and had pronociceptive actions per se [41,58,60,61]. Ligands of the dopamine D1 receptor (D1R) injected into the dorsal or ventral striatum did not influence acute hyperalgesia induced by formalin or prostaglandin E2 [60,61]. These observations suggest that dopamine acting within the dorsal and ventral striatum exerts an antinociceptive effect, which is mediated by the D2R but apparently not the D1R.

## 7. Chronic Pain as a Hypodopaminergic State

A considerable body of evidence now suggests that chronic pain is associated with reduced dopaminergic neurotransmission in the mesostriatal pathways. Most of the animal studies concerning this issue have focused on the mesolimbic system (VTA-NAc) despite the existing evidence that the nigrostriatal pathway is also involved in pain processing (see references to the dStr and SNc in Section 3, Section 4, Section 5 and Section 6). Thus, it was found that chronic neuropathic or inflammatory pain was accompanied by reduced basal extracellular dopamine levels and tissue dopamine levels in the NAc, as well as a diminished rate of dopamine metabolism [62,63,64,65]. Increased NAc level of the dopamine transporter (DAT) was observed concomitantly, suggesting augmented dopamine reuptake [63,65]. Moreover, changes in electrophysiological properties of dopaminergic neurons in the VTA were reported: their intrinsic excitability and spontaneous firing rate were reduced in models of neuropathic or cancer pain [53,63,66,67]. Studies in humans and animals also revealed attenuated dopaminergic responses to noxious and rewarding stimuli. Patients suffering from fibromyalgia or chronic back pain showed blunted release of dopamine in the dorsal and ventral striatum in response to painful stimulation [33,68]. A decrease in systemic morphine-induced dopamine release in the NAc was observed in rats with neuropathy, and attenuation of opioid and psychostimulant drug reward, strictly dependent on dopamine, was demonstrated using the CPP paradigm in models of neuropathic and inflammatory pain [69,70].

Given the essentially antinociceptive actions of mesostriatal dopamine, these changes in the dopamine system activity might be regarded as potential contributing factors to the development and maintenance of chronic pain. However, recent research using refined techniques, which allow the targeting of strictly defined neuronal populations, suggests that the picture may be more complicated, strongly depending on the striatal subregion and localization of its afferent dopaminergic cells [66,67] (see Section 10.3 and Section 10.4).

## 8. Striatal Projection Neurons and the Effect of Dopamine on the Excitatory Inputs to the Striatum

The predominant mechanism by which dopamine affects striatal output is by postsynaptic modulation of glutamatergic neurotransmission. The striatum, composed mainly of GABAergic projection neurons, is densely innervated by glutamatergic afferents originating in the cerebral cortex, hippocampus, amygdala, and thalamus [20]. Two intermixed populations of striatal projection neurons (medium spiny neurons—MSN) are distinguished, which differ largely in terms of their projection targets, dopamine receptor expression, and neuropeptide content. The D1-MSN (also called the direct pathway striatal projection neurons—dSPN) express the D1 dopamine receptor as well as the neuropeptides dynorphins and substance P. The D2-MSN (also called the indirect pathway striatal projection neurons—iSPN) express the D2 dopamine receptor and the neuropeptides enkephalins [71]. D1-MSN of the dStr project to the substantia nigra pars reticulata and the entopeduncular nucleus (via the so-called direct pathway), whereas the dorsal striatal D2-MSN project to the globus pallidus (via the indirect pathway) [71,72,73]. Outputs from the NAc are less segregated: while D1-MSN project to the VTA, both D1- and D2-MSN project to the ventral pallidum [20,74,75]. The NAc, compared to dStr, also contains a higher proportion of MSN co-expressing both dopamine receptors or neuropeptides characteristic of both canonical types of striatal MSN [76,77].

D1 and D2 dopamine receptors, both belonging to G protein-coupled receptors, exert opposite effects on excitatory neurotransmission within the striatum due to their different G protein coupling. The D1 receptor, coupled to the G_olf_ protein, increases MSN excitability, thus facilitating glutamatergic input to the direct pathway. In contrast, the D2 receptor, coupled to the G_i/o_ proteins, decreases MSN excitability, hampering glutamatergic neurotransmission onto the indirect pathway [78].

The mechanisms by which these regulatory actions occur are very complex and involve the effects of second messengers and protein kinases on voltage-dependent ion channels and glutamate receptor channels expressed by the MSN, as reviewed in [73,78]. Thus, stimulation of the D1 receptor leads to activation of adenylyl cyclase by the G_olf_ protein α subunit, resulting in cAMP synthesis and activation of the cAMP-dependent protein kinase A (PKA). Stimulation of that signaling pathway by dopamine produces an increase in cell surface expression of the AMPA and NMDA glutamate receptors, phosphorylation of the dendritic L-type voltage-dependent Ca^2+^ channels (containing the Cav1.3 subunit), which enhances their opening, increases the Ca^2+^ ion influx into neurons, and also augments cation influx via the NMDA receptors (cf [73,78]). All these PKA-dependent effects increase the responsiveness of the D1-MSN to glutamate following D1 receptor activation.

On the other hand, the D2 receptor is coupled to the G_i_ and G_o_ proteins. The G_i_ protein α subunit inhibits adenylyl cyclase, whereas the βγ subunit complexes stimulate phospholipase Cβ leading to the production of diacylglycerol, which activates protein kinase C (PKC), and of inositol 1,4,5-triphosphate, which elicits the release of Ca^2+^ from intracellular stores. Activation of the D2 receptor by dopamine produces a decrease in cell membrane expression of the AMPA receptors due to dephosphorylation of the GluR1 subunit, reduces the opening of the L-type voltage-dependent Ca^2+^ channels (via a Ca^2+^-calmodulin-dependent mechanism), reduces the opening of voltage-dependent Na^+^ channels (by a mechanism involving PKC) and enhances opening of Kir3 K^+^ channels (cf [73,78]). All the above-mentioned effects of the D2 receptor stimulation on voltage- and glutamate-gated ion channels reduce the responsiveness of the D2-MSN to glutamate. In addition, stimulation of the striatal D2 receptor reduces the release of glutamate itself [79].

## 9. Involvement of Medial Prefrontal Cortex in Pain Processing

Among the glutamatergic inputs to the striatum, the projections from the mPFC to the NAc seem particularly relevant to pain, according to the current evidence. Using functional magnetic resonance imaging in humans suffering from chronic back pain, Baliki, Apkarian, and colleagues demonstrated that functional connectivity between the mPFC and NAc, as well as activity of the mPFC, reflect the intensity of pain [27,80]. In a longitudinal study of progression from subacute to chronic back pain, these authors also showed that the mPFC-NAc functional connectivity was stronger in those patients who did progress to chronic pain after a subacute episode than in those who did not. Moreover, the initial strength of this connection was predictive of pain chronification (i.e., the transition from subacute to chronic pain) [24]. Connectivity changes between the NAc and mPFC in chronic pain sufferers were also reported by Makary et al. [26].

mPFC encompasses the infralimbic and prelimbic cortex (IL, PL), and the adjacent anterior cingulate cortex (ACC). These distinct parts of the mPFC play different roles in pain modulation: activation of the PL glutamatergic neurons reduces sensory and affective pain responses in neuropathic pain [81,82,83], whereas activation of the ACC exerts an opposite effect, particularly on the emotional aspect of pain, i.e., mediates or enhances its aversive quality [84,85,86,87]. According to most studies, the ACC does not modulate the sensory experience of pain; however, some results suggest that ACC stimulation may also enhance sensory sensitivity [84,85,88].

**Figure 1:**
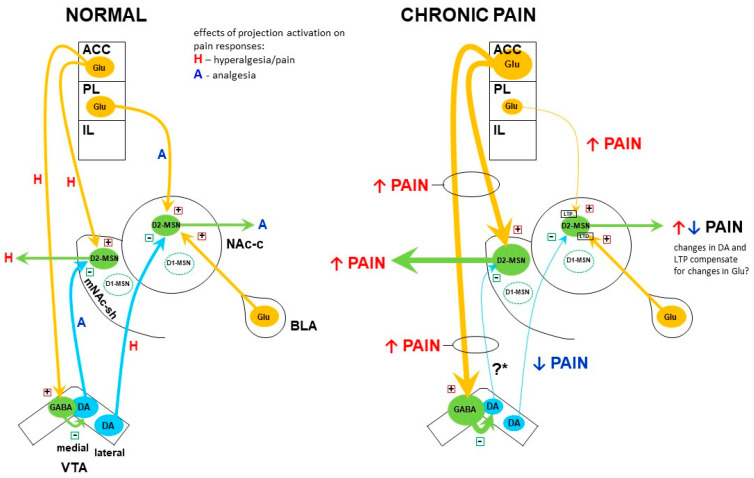
Interactions between the Ventral Mesostriatal Dopaminergic Pathway and Corticostriatal Glutamatergic Afferents in Chronic Pain. The scheme presents only those neuronal connections, whose participation in pain processing and involvement in chronic pain have been documented directly. Glutamatergic, dopaminergic, and GABAergic neurons are depicted as orange, blue, and green ovals, respectively, and their projections are depicted as arrows. “+” and “−“ signs denote excitatory and inhibitory effects on the postsynaptic cell, respectively. **Left panel.** “A” and “H” placed along projections denote effects of *activation* of the particular projection on pain responses: analgesia and hyperalgesia/pain, respectively. These signs reflect effects on both sensory and affective aspects of pain, or either (see text for details), and show general tendencies, some of which become explicit only under tonic or chronic pain. **Right panel.** Changes in the activity of particular neuronal populations and their projections under chronic pain have been reflected by the “cell body” size and the arrow width. ↑ and ↓ arrows denote the final effect on pain (increase and decrease, respectively) resulting from both the function of a particular projection in pain modulation (refer to “A” and “H” on the left panel) and changes in the activity of this projection in chronic pain. ?*–In contrast to some other studies, Ren et al. [67] provide evidence of an *increase* in dopaminergic neurotransmission in the medial VTA–medial NAc shell projection in chronic pain. This scheme has been based on the studies by Schwartz et al. [89], Lee et al. [81], Ren et al. [63,67], and Gao et al. [85]. The nigrostriatal dopamine pathway has not been included in the figure due to the lack of corresponding studies on the dorsal striatum. ACC—anterior cingulate cortex; BLA—basolateral amygdala; D1-MSN—D1 receptor-expressing medium spiny neuron; D2-MSN—D2 receptor-expressing medium spiny neuron; DA—dopamine; IL—infralimbic cortex; LTD—long-term depression; LTP—long-term potentiation; mNAc-sh—medial nucleus accumbens shell; NAc-c—nucleus accumbens core; PL—prelimbic cortex; VTA—ventral tegmental area.

Figure 1 shows a schematic representation of the interactions between the mesostriatal and corticostriatal systems, and their changes in chronic pain as described below in Section 9 and Section 10.

In chronic pain states, the activity of PL is decreased [82,83,90,91] (see also the review by Thompson and Neugebauer [17]), whereas the ACC is overactive [92,93,94]. Taking into account the role of each mPFC subdivision in pain processing, these changes may be regarded as supporting pain, and normalization of the PL and ACC activity is expected to confer analgesia (cf [17] and the references in the previous paragraph). While the above picture of the PL and ACC involvement in pain generally holds true, new studies using refined techniques reveal additional intricacies, e.g., opposite modulation of pain aversion by caudal vs. rostral ACC, and by the afferents to ACC from the mediodorsal thalamus vs. those from the basolateral amygdala [95,96]. Regarding the PL activity regulation in chronic pain, Mitrić et al. [97] demonstrated a decrease in excitability of glutamatergic neurons only in cortical layer 5, whereas excitability of cells in layer 2/3 was increased during painful neuropathy in mice.

The role of IL in pain is even less straightforward. David-Pereira et al. [98] reported that IL stimulation (with glutamate) or inactivation (using local anesthetics) affected nociception, which indicates that the IL is involved in pain processing. Inactivation of the IL had a similar effect as inactivation of the PL, namely facilitated nociception, implying that the IL, like PL, exerted a tonic antinociceptive effect, both in acute and chronic pain. However, the effects of excitatory stimulation of each mPFC region differed, stimulation of the PL conferring fast analgesia, and activation of the IL having a slow onset pronociceptive effect mediated by a metabotropic glutamate receptor (mGluR5) [98]. On the other hand, the study by Jiang et al. (2014) demonstrated that the IL, in contrast to PL, does not mediate pain aversion in a formalin-induced conditioned place avoidance model. A change in the IL activity was reported in a model of chronic inflammatory pain, namely a decrease resulting from enhanced feedforward inhibition by amygdala neurons [99]; in contrast to the observations by David-Pereira et al. [98], Kiritoshi and colleagues [99] found that restoring the IL activity and output inhibited pain behaviors and pain-related cognitive deficits via activation of mGluR5. On the other hand, no changes in the IL pyramidal neurons excitability (again, in contrast to PL) were detected during neuropathic pain, even though changes in the morphology of their dendritic arbors were reported [97]. Some opposing effects of IL vs. PL might result from inhibition of the PL pyramidal neurons by glutamatergic input from the IL [100].

Altogether, the above evidence shows that the PL activity inhibits pain and that it is reduced in chronic pain (at least in cortical layer 5), whereas the ACC enhances pain (at least in the emotional-aversive aspect) and the ACC activity is increased in chronic pain. The IL does modulate pain processing but in ways that still remain unclear.

## 10. Interactions of the Dopamine System with mPFC Projections to the NAc in Pain Regulation

### 10.1. Medial NAc Shell

Pharmacological studies involving injections of dopamine receptor ligands into the dStr and NAc suggest that stimulation of the D2R exerts an analgesic effect, whereas the striatal D1R plays no role in pain (see Section 6). Moreover, numerous studies demonstrated that chronic pain selectively affects the D2-MSN’s (but not D1-MSN’s) dendrite morphology, excitability, and synaptic connectivity [63,67,85,89]. This implicates the indirect striatal output pathway, stemming from the D2R-containing MSN, rather than the direct pathway originating in D1-MSN, in pain processing. Since dopamine acts in the striatum to reduce pain, and it inhibits the indirect pathway via stimulation of the D2R, it might be assumed that activation of the indirect pathway is pronociceptive, whereas its inhibition reduces pain. Indeed, Ren et al. [63] demonstrated that direct chemogenetic stimulation of D2-MSN in the mNAc shell enhanced tactile allodynia, while chemogenetic inhibition of these neurons reduced allodynia, in a mouse model of neuropathic pain.

These authors also showed that the excitability of the D2-MSN (but not D1-MSN) was increased in animals with neuropathy. That change apparently resulted from a reduction in dopaminergic neurotransmission (due to decreased VTA dopamine neurons firing and increased dopamine reuptake) and was reversed by dopaminomimetic drugs [63]. Therefore, the study by Ren et al. [63] suggested that the hypodopaminergic state accompanying neuropathic pain enhanced the stimulatory effect of glutamate on the indirect pathway MSN in the mNAc shell, thus aggravating tactile hypersensitivity.

Interestingly, the strength of the main cortical excitatory input to this part of the NAc, namely from the IL, was reduced in mice with neuropathy [63], which might be considered as an adaptive change limiting cortically driven activation of the D2-MSN and thus limiting pain.

The mNAc shell was also identified as a target of projections from the ACC in the recent study by Gao et al. [85]. These authors found that the mNAc shell-projecting ACC glutamatergic neurons became hyperexcitable in a rat model of neuropathic pain. Their chemogenetic inhibition did not influence sensory hypersensitivity elicited by neuropathy. However, inhibition of these neurons induced CPP in rats with neuropathy (but not in control rats experiencing no pain), which suggests that overactivity of the ACC–mNAc shell projection contributes to chronic pain-related aversion. Furthermore, Gao et al. [85] demonstrated that the ACC–NAc projection targeted selectively D2-MSN (rather than D1-MSN) in the mNAc shell and inhibition of this neuronal population reduced aversion elicited by activation of the NAc input from the ACC. Thus, the study by Gao et al. [85] indicates that chronic pain-related aversion depends on the overactivation of the indirect pathway MSN in the mNAc shell by the input from the ACC.

The same study also implicates the projections from the ACC to the VTA in pain aversion [85]. The authors demonstrated that axons of the ACC excitatory neurons selectively targeted GABAergic (rather than dopaminergic) neurons in the rostral medial VTA. Like in the NAc, silencing these projections induced CPP in animals with neuropathy but did not restore normal pain thresholds. Inhibition of GABAergic signaling within the VTA by a local administration of a GABAA receptor antagonist blocked aversion elicited by enhanced input from the ACC. These results suggest that, in chronic pain, stimulation of the GABAergic neurons in the VTA by the overactive input from the ACC contributes to the pain-related aversion but not sensory hypersensitivity [85].

GABAergic cells in the VTA inhibit dopaminergic neurons. Thus, the enhanced activity of GABAergic cells may lead to a decrease in dopaminergic neurons activity, which was indeed observed in chronic pain states [53,63,66,67]. This may cause disinhibition of the D2-MSN in the mNAc shell (via the D2 receptor), further adding to the overactivity of the NAc output via the indirect pathway. Therefore, it appears that the ACC may mediate the aversiveness of chronic pain by stimulating the indirect output pathway from the mNAc shell, both via the direct glutamatergic corticostriatal projection and via the indirect route involving reduced inhibition of D2-MSN by mesostriatal dopamine (Figure 1).

While the study by Gao and colleagues [85] implicated the projection from the ACC to the mNAc shell D2-MSN only in the affective but not sensory aspect of pain, Ren et al. [63] demonstrated that stimulation or inhibition of D2-MSN in the same NAc region did affect allodynia in mice with neuropathy. The discrepancy between these findings may have resulted from species differences (rats vs. mice were used), as well as from methodological differences in identifying and targeting the D2-MSN (MSN expressing the D2R vs. MSN expressing the adenosine A2A receptor; both neuronal populations show high overlap but are not strictly identical). Consequently, those two research groups may have studied slightly different MSN subpopulations, which may also be distinct in terms of inputs and outputs in rats as compared to mice.

### 10.2. NAc Core

Largely opposite relationships were described by Lee et al. [81] regarding the projection from the PL to the NAc core. Optogenetic stimulation of glutamatergic neurons in the PL or their terminals in the NAc core increased nociceptive thresholds in intact rats and reduced sensory hypersensitivity in animals with neuropathic pain. Such stimulation also induced CPP and normalized chronic pain-related changes in sucrose preference as well as in the forced swim test (i.e., the decreased preference and the increased immobility time, respectively) [81]. These results indicate that the PL projection to the NAc core regulates both sensory sensitivity and affective symptoms of chronic pain such as aversion, anhedonia, and behavioral despair. Since the PL activity is reduced in chronic pain [82,83,90,91], the above findings suggest that deficiency in the excitatory PL input to the NAc core may contribute to the maintenance of pain in its sensory and affective aspects. However, the effects of PL hypoactivity may be counterbalanced by postsynaptic changes, since Ren et al. [67] demonstrated that the PL synapses on the D2-MSN of the NAc core were strengthened in a model of neuropathy (Figure 1).

On the other hand, a long-term depression (LTD) of excitatory synapses on the D2-MSN in the NAc core was also reported in models of inflammatory and neuropathic pain [89]. It was due to the upregulation of the NMDA receptor GluN2B subunits and was causally linked to the chronic pain-related loss of motivation to work for a natural reward [89]. As suggested by the findings by Ren et al. [67], this form of LTD takes place selectively on synapses innervated by the basolateral amygdala but not the PL. 

### 10.3. Contrasting Effects of Dopamine in the Medial NAc Shell vs. NAc Core in Pain Modulation

Interestingly, all changes in the strength of excitatory synapses detected in the NAc core in chronic pain states occurred selectively in D2-MSN, and not in D1-MSN [67,89]. This resembles the selectivity towards modifications of the indirect rather than the direct pathway MSN in the mNAc shell in chronic pain [63,85]. However, the D2-MSN in those two parts of the NAc appear to exert different effects on pain symptoms. Direct chemogenetic activation of the D2-MSN in the NAc core reversed neuropathic pain-related emotional deficits (anxiety and reduced social interactions) but did not influence sensory sensitivity. These effects were mimicked by chemogenetic inhibition of identified VTA dopaminergic neurons which project to the NAc core-the manipulation which disinhibits the D2-MSN (by relieving the dopamine effect on the D2R) [67]. In contrast, chemogenetic activation of the D2-MSN in the mNAc shell enhanced allodynia in mice with neuropathic pain, whereas inhibition of these neurons reduced allodynia, as did chemogenetic activation of dopaminergic neurons which innervate them [63,67].

Taken together, these observations suggest that the NAc core D2-MSN reduce pain (in its affective dimension), whereas the mNAc shell D2-MSN exacerbate pain (in its sensory dimension). Dopamine, which inhibits the D2-MSN in both parts of the NAc, influences pain symptoms in a reverse manner: it has a proalgesic effect in the NAc core (at least in terms of some affective symptoms) and an analgesic effect in the mNAc shell [63,67] (Figure 1). Notably, the NAc core and mNAc shell are innervated by different populations of dopamine neurons: the NAc core is innervated by projections from the lateral VTA, and the mNAc shell by projections from the medial VTA [18,19,20]. Moreover, D2-MSN located in either NAc subregion project to distinct parts of the ventral pallidum (VP; dorsolateral vs. medial, respectively; cf [18]). All in all, it appears that pain is processed in the basal ganglia by two separate circuits playing contrasting roles: the pain-relieving lateral VTA–NAc core–dorsolateral VP pathway, and the pain-enhancing medial VTA–mNAc shell–medial VP pathway. (Interestingly, Ikemoto [18] also pointed to disparate roles of these two systems in the context of reward mechanisms). As shown in Figure 1, the above-mentioned circuits might be regulated independently by inputs from separate parts of the mPFC. However, it has to be recognized that the figure presents only those corticostriatal projections whose involvement in pain modulation has been studied thoroughly, whereas no comparable data could be found concerning the known projections from the IL to the mNAc shell and from the ACC to the NAc core [20,101].

Regarding the main output target of the ventral striatal D2-MSN, the VP, there are few studies on its role in pain. Nevertheless, analgesic effects of opioids injected into the pallidum, including the VP, were reported [102]. What is important, the opioid peptides enkephalins are present in large amounts in axon terminals of the D2-MSN projecting from the NAc to the VP, and they regulate GABAergic neurotransmission therein [103]. The above observations support the view that the VP participates in pain modulation and suggest the involvement of enkephalins expressed in the ventral striatopallidal pathway in these processes.

### 10.4. Alterations in the Dopamine System and Their Role in Chronic Pain—A Reappraisal

As discussed in Section 7, an overwhelming body of evidence indicates that dopaminergic neurotransmission in the VTA–NAc pathway is deficient in chronic pain states. According to the predominant view, this deficiency maintains and exacerbates pain. However, such a general view is not consistent with the findings that dopamine actually promotes pain when acting in the NAc core, in contrast to the mNAc shell. Therefore, it seems that functional changes in the dopamine system during chronic pain and their impact on pain should be considered separately for each subregion of the NAc.

Studies by Huang et al. [66] and Ren et al. [67] suggest that dopamine neurons in the lateral VTA, which project to the NAc core, are inhibited during neuropathic pain. The latter authors also show that such inhibition reduces some emotional symptoms associated with pain. Thus, Ren et al. [67] argue that the decrease in dopaminergic signaling in the lateral VTA–NAc core projection is a compensatory change rather than a maladaptive alteration underlying pain.

In their 2021 study, Ren et al. [67] also presented indirect evidence indicating that neurotransmission in the projection from the medial part of the VTA to the mNAc shell is enhanced rather than reduced in chronic pain. The authors interpreted this finding as another adaptive change resulting in reduced pain intensity (diminished allodynia). In line with their observation, Huang et al. [66] described an increase in excitability of a subpopulation of medial VTA dopamine neurons, while Sagheddu et al. [104] detected increased burst firing in some VTA dopamine cells and increased extracellular dopamine level in the NAc shell in models of painful neuropathy. However, these findings are difficult to reconcile with another study demonstrating reduced spiking of dopamine neurons in the medial VTA and decreased extracellular dopamine levels in the mNAc shell in a similar model of pain [63]. Moreover, rodents with neuropathy show attenuated mesostriatal response to morphine, the drug which acts (indirectly) by depolarizing dopamine neurons. During chronic pain, morphine-elicited dopamine release in the NAc is dampened, resulting in a reduction of the drug’s rewarding effect in the CPP paradigm [69,70]. This suggests a decrease rather than an increase in dopamine neurons activity, which leads to a blunted behavioral response to a rewarding stimulus, reflecting chronic pain-related anhedonia. Therefore, alterations of the activity in the medial VTA–mNAc shell projection in chronic pain remain an open question.

## 11. Altered Modulation of Dopamine Release in Chronic Pain

Dopaminergic activity in the dStr/NAc may also be regulated at the level of neurotransmitter release. Mesostriatal dopaminergic axon terminals express several types of inhibitory receptors, including the D2R autoreceptor and the kappa opioid receptor (KOR), whose stimulation hampers the release of dopamine [105,106,107]. These receptors are also present on dopamine neuron somata, where they reduce cell excitability. Some studies show that the presynaptic D2R function might be suppressed in the NAc in chronic pain, and the level of the D2R transcript is reduced in the midbrain dopamine neurons [108,109]. This suggests that autoinhibitory control of dopamine over its neurons may be limited during persistent pain, which could be regarded as a compensatory mechanism, acting to restore the dopamine system function in cases of its deficiency. A similar net effect could be produced by up-regulation of the postsynaptic dopamine receptors in the NAc. Indeed, increased levels of D2R mRNA were detected in the NAc in some studies [109,110] but others demonstrated a decrease or no change [22,104]. This lack of consistency may result from opposite changes in dopamine levels in different NAc subregions, as suggested by Ren et al. [67], and from different durations of pain in particular experiments [22].

Less ambiguous data have been reported regarding the role of the presynaptic KOR in chronic pain. Stimulation of this receptor located on dopaminergic axon terminals in the NAc is known to produce aversion due to inhibition of dopamine release [111,112,113]. The striatum, and particularly the NAc shell, contains high levels of dynorphins, synthesized by the D1-MSN, which act as KOR agonists [113,114]. In models of chronic pain, the elevation of the prodynorphin gene expression and the dynorphin peptide level, as well as increased occupancy of KOR by endogenous agonists were reported in the NAc [109,115,116]. Enhanced presynaptic KOR expression and activity were also demonstrated: during neuropathic and/or inflammatory pain, the KOR mRNA level was increased in the VTA, and agonist-stimulated coupling of KOR to G-proteins was elevated both in the VTA and NAc [115,116]. These observations provide evidence that the dynorphin–presynaptic KOR signaling is enhanced in the NAc in chronic pain states due to up-regulation of both KOR and its endogenous agonist dynorphin. Taking into account that activation of this signaling system produces aversion in healthy animals, its chronic overactivity in models of persistent pain is likely to contribute to pain-associated affective disturbances. Indeed, it was demonstrated that inhibiting the dynorphin action in the NAc by KOR antagonism reversed a pain-related motivational deficit and produced CPP in animals with chronic pain [115,116]. According to the authors’ interpretation, the CPP was caused by relief from the chronic aversive state associated with pain because KOR antagonism did not produce CPP in pain-free control animals [115]. In addition, studies showed that infusions of KOR antagonists or an antibody against dynorphin reversed opioid reward deficits and loss of the underlying dopamine release in rodents with chronic pain [115,117,118]. Some of the above-mentioned studies demonstrated the effects of KOR antagonists or an anti-dynorphin antibody after their administration directly into the NAc, in particular to the NAc shell [116,117]. On the other hand, KOR antagonists did not affect chronic pain-related hyperalgesia [116].

Thus, these studies provide convincing evidence that affective disturbances associated with chronic pain, such as aversion, diminished motivation, and a deficit in drug reward, are caused, at least in part, by enhanced signaling in the dynorphin–KOR system within the NAc. These effects appear to be underlain by the presynaptic KOR-mediated inhibition of dopamine release in the NAc shell.

## 12. Summary

The basic view of chronic pain as a hypodopaminergic state emerged from experiments involving stimulation or lesions of the mesencephalic dopaminergic neurons, injections of dopamine receptor ligands into the dStr and NAc in rodent models of pain, as well as human brain imaging studies in chronic pain conditions and observations of pain symptom profiles in PD. According to this view, dopaminergic neurotransmission is diminished during chronic pain and this deficiency contributes to the maintenance of the sensory and/or affective symptoms of pain, whereas enhancing dopamine signaling, e.g., by administration of dopamine agonists, produces analgesia via stimulation of the D2R in the dStr and NAc [119,120].

The subsequent use of novel techniques involving transgenic animals, opto- and chemogenetics, gave unprecedented insights into the complexities of the mesostriatal pathways and their interactions with the corticostriatal excitatory input in the context of pain. Studies using these new methods have so far yielded the major finding of two separate mesolimbic dopaminergic projections which exert opposite effects on pain and interact with separate inputs from the mPFC (Figure 1). One of these circuits is made up of dopaminergic neurons of the medial VTA which innervate the mNAc shell and interact with glutamatergic afferents from the ACC. Dopamine acting within this circuit produces analgesia by inhibiting the D2-MSN of the mNAc shell. The second circuit is made up of dopaminergic neurons of the lateral VTA which project to the NAc core, where they interact with glutamatergic afferents from the PL. Dopamine in this circuit enhances pain by its inhibitory effect on the D2-MSN of the NAc core [63,67,81,85,89]. Whereas there are some inconsistencies between the findings regarding the specific roles of each circuit in modulating the sensory vs. affective aspects of pain, the compiled evidence suggests the involvement of each circuit in both aspects.

Importantly, alterations in the dopamine system activity in specific projections and their impact on chronic pain appear more nuanced than previously thought. Although (in agreement with the basic view) there is a reduction of dopaminergic neurotransmission in the lateral VTA–NAc core projection, this change seems to reduce (affective) pain symptoms rather than enhance pain [67]. Regarding the medial VTA–mNAc shell projection, some studies suggest a reduction in dopaminergic transmission therein, which exacerbates chronic pain (in agreement with the basic view); however, other reports suggest the opposite: that this pathway is overactive during pain, which plays a protective role (see Section 10.4). On the other hand, there is a striking lack of detailed studies on the nigrostriatal system in chronic pain, even though previous research clearly indicated its engagement.

## 13. Methodological Issues

As already alluded to, some findings regarding the involvement of the dopamine system in pain mechanisms seem incongruent with one another. These discrepancies are most likely due to (sometimes major) differences in experimental paradigms applied in the studies.

(1) There are large differences in the duration of pain in “chronic” pain animal models. In most cases, pain duration does not exceed two weeks, and in many models, the pain lasts only a few days, whereas in humans, pain is defined as chronic when it lasts for longer than three months (or even six months) [121,122]. This raises the possibility that various experimental paradigms are not equivalent in reflecting particular stages of chronic pain development and maintenance. Chang and colleagues [22] demonstrated directly that alterations in the ventral mesostriatal system develop gradually as the pain continues and differ markedly between animals subjected to pain for five days vs. four weeks. Dias et al. [60], in turn, showed that during the transition from acute/tonic to chronic pain, there is a shift from antinociceptive to pronociceptive effect in the accumbal dopamine action on pain. On the other hand, the work by Vergara et al. [123] suggested that the ventral mesostriatal dopamine is involved in the mechanism of pain chronification but not in the maintenance of already established chronic pain. Whereas the results by Dias et al. [60] and Vergara et al. [123] are not consistent with other reports ([124]; cf also Section 5 and Section 6), perhaps due to the use of a peculiar model, they do draw attention to the fact that the modulatory effect of dopamine on pain may change as pain progresses. However, in many other studies, measurements were performed only at a single time-point and thus provide just a snapshot of dynamic changes, which may also evolve at different paces depending on the model. The changes in the mesostriatal dopaminergic activity and the dopamine–glutamate interactions described in Section 10 of this review are based on findings in models of neuropathic pain (due to sciatic nerve lesion) of five to fourteen days duration. In order to determine whether they represent the subacute, transitory, or chronic pain stage, it would be worthwhile to repeat some key experiments at much more delayed time-points, e.g., two-three months.
(2) Different species (rats and mice) and strains thereof are used in the studies. While that is dictated mostly by methodological reasons (e.g., availability of transgenic mouse strains), biological differences between these organisms may be a cause of some discrepancies in pain studies. The more detailed the studies (e.g., getting down to particular subsets of projection fibers between small brain subregions), the more impact the species/strain factor is likely to have on the outcomes. Morphological differences in the dopamine system were demonstrated between inbred strains of mice, including distinct dopaminergic fibers distribution within the NAc shell [125]; even more dissimilarities in brain connectivity might be expected between mice and rats, not to mention humans.
(3) The studies focus on increasingly small subregions of brain structures. When comparing data from different laboratories, it is sometimes difficult to decide if the same or distinct subregions were analyzed. For example, Ren and colleagues [63] studied dopamine effects on pain in the medial NAc shell, whereas the study by Massaly et al. [116] focused on the *ventro*medial NAc shell. The first study found effects on allodynia, whereas the second one excluded effect on the sensory dimension of pain but found a role of a corresponding manipulation on pain affect. It is not entirely clear if the results were dissimilar because different parts of the NAc shell were studied or because of other factors.
(4) Different gene promoters are used in transgenic animals to target neuronal populations that we tend to think of as one population. In the striatum/NAc, there is the basic distinction between the two major populations of projection neurons, the indirect pathway- and the direct pathway-MSN. The D2R, adenosine receptor A2A, and proenkephalin are typical markers of the indirect pathway MSN, and promoters of these three genes are treated somewhat interchangeably to target the “D2-MSN”. However, as mentioned in Section 10.1, there is no 100% co-expression between the D2R, A2A receptor, and proenkephalin; thus, experimental manipulations on each cell population may yield different results (e.g., [126]). In the indirect pathway MSN, the D1R and prodynorphin are the analogous set of markers.

## 14. Concluding Remarks

The accumulated evidence clearly supports the major role of the mesostriatal system in the modulation of chronic pain symptoms and the emergence of functionally relevant alterations within that system in the course of pain progression. Inconsistencies in the details of different studies likely reflect the interplay of adaptive vs. maladaptive changes which develop as pain persists for some time and lead either to its quenching or consolidation. This is illustrated well by the paper by Ren et al. [67], who report homeostatic changes in the internal excitability of the NAc D2-MSN, which counteract the functionally adaptive (i.e., pain-diminishing) changes in the activity of the VTA–NAc medial and lateral pathways. 

Another form of delayed compensatory plasticity was described by Wang and colleagues [127,128]. They found that the GluA1 subunits of the AMPA receptor were up-regulated in the NAc synapses (both in the core and shell) during chronic inflammatory and neuropathic pain, which resulted in the formation of calcium-permeable AMPA receptors lacking the GluA2 subunit. By using specific ligands of these receptors with altered subunit composition, the authors demonstrated that their appearance limited chronic pain-related anhedonia [127,128]. Nevertheless, anhedonia was clearly present at the time when the GluA1 subunits were enriched in the synapses, which indicates that the adaptive effect of GluA1 up-regulation was overcome by a stronger mechanism sustaining the depressive-like quality of pain. Thus, lasting pain is a dynamic process, which includes both compensatory and maladaptive changes in the mesostriatal system evolving in time. More time-course studies are required in order to determine which of them actually prevail and contribute to the sensory and affective symptoms of pain in the long term.

From a practical standpoint, the idea of chronic pain as a hypodopaminergic state generally holds true and draws attention to the potential usefulness of dopaminomimetic drugs in the treatment of chronic pain conditions. The efficacy of non-specific psychostimulants such as amphetamine and methylphenidate has been confirmed in clinical settings in patients suffering from postoperative or cancer pain, where they potentiated analgesia and diminished side effects produced by opioid agonists ([129,130]; see also the review by Dalal and Melzack [131]). While the usefulness of these particular drugs is doubtful due to their strong addictive potential, dopamine receptor agonists, e.g., pramipexole with its moderate reinforcing value [132], show more promise. In fact, pramipexole was found effective in the treatment of fibromyalgia [133], whereas case studies also reported successful treatment of burning mouth syndrome and thalamic pain with pramipexole or apomorphine [134,135]. Finally, studies in animal models indicate specific changes in the subunit composition of the NMDA and AMPA receptors which take place in the NAc during persistent pain and influence the affective dimension of pain [89,127]. Selective ligands of those altered glutamate receptors could be considered as a potential treatment in chronic pain conditions.
